# Biomarkers and computational models for predicting efficacy to tumor ICI immunotherapy

**DOI:** 10.3389/fimmu.2024.1368749

**Published:** 2024-03-08

**Authors:** Yurong Qin, Miaozhe Huo, Xingwu Liu, Shuai Cheng Li

**Affiliations:** ^1^ Department of Computer Science, City University of Hong Kong, Kowloon, China; ^2^ City University of Hong Kong Shenzhen Research Institute, Shenzhen, Guangdong, China; ^3^ School of Mathematical Sciences, Dalian University of Technology, Dalian, Liaoning, China

**Keywords:** tumor, ICI immunotherapy, biomarkers, computational models, prediction of treatment effectiveness

## Abstract

Numerous studies have shown that immune checkpoint inhibitor (ICI) immunotherapy has great potential as a cancer treatment, leading to significant clinical improvements in numerous cases. However, it benefits a minority of patients, underscoring the importance of discovering reliable biomarkers that can be used to screen for potential beneficiaries and ultimately reduce the risk of overtreatment. Our comprehensive review focuses on the latest advancements in predictive biomarkers for ICI therapy, particularly emphasizing those that enhance the efficacy of programmed cell death protein 1 (PD-1)/programmed cell death-ligand 1 (PD-L1) inhibitors and cytotoxic T-lymphocyte antigen-4 (CTLA-4) inhibitors immunotherapies. We explore biomarkers derived from various sources, including tumor cells, the tumor immune microenvironment (TIME), body fluids, gut microbes, and metabolites. Among them, tumor cells-derived biomarkers include tumor mutational burden (TMB) biomarker, tumor neoantigen burden (TNB) biomarker, microsatellite instability (MSI) biomarker, PD-L1 expression biomarker, mutated gene biomarkers in pathways, and epigenetic biomarkers. TIME-derived biomarkers include immune landscape of TIME biomarkers, inhibitory checkpoints biomarkers, and immune repertoire biomarkers. We also discuss various techniques used to detect and assess these biomarkers, detailing their respective datasets, strengths, weaknesses, and evaluative metrics. Furthermore, we present a comprehensive review of computer models for predicting the response to ICI therapy. The computer models include knowledge-based mechanistic models and data-based machine learning (ML) models. Among the knowledge-based mechanistic models are pharmacokinetic/pharmacodynamic (PK/PD) models, partial differential equation (PDE) models, signal networks-based models, quantitative systems pharmacology (QSP) models, and agent-based models (ABMs). ML models include linear regression models, logistic regression models, support vector machine (SVM)/random forest/extra trees/k-nearest neighbors (KNN) models, artificial neural network (ANN) and deep learning models. Additionally, there are hybrid models of systems biology and ML. We summarized the details of these models, outlining the datasets they utilize, their evaluation methods/metrics, and their respective strengths and limitations. By summarizing the major advances in the research on predictive biomarkers and computer models for the therapeutic effect and clinical utility of tumor ICI, we aim to assist researchers in choosing appropriate biomarkers or computer models for research exploration and help clinicians conduct precision medicine by selecting the best biomarkers.

## 1 Introduction

In the last few years, tumor immunotherapy research has advanced rapidly due to the emergence of innovative therapies such as immune checkpoint inhibitor (ICI) immunotherapy, cell immunotherapy, and cancer vaccine (1). Compared to conventional treatments, tumor immunotherapy has exhibited longer-lasting effects (2–4). ICI therapy is a leading research direction of tumor immunotherapies, with the most representative ICI being programmed cell death protein 1 (PD-1)/programmed cell death-ligand 1 (PD-L1) inhibitors and cytotoxic T-lymphocyte antigen-4 (CTLA-4) inhibitors. These inhibitors restart the human immune system to fight cancer, such as liver, lung, colon, breast, skin, etc. (5). Yet, only a subset of patients achieves enduring efficacy and survival with ICI therapy. Determining biomarkers that effectively identify patients that will benefit from ICI would minimize unnecessary therapy costs and severe toxicity for the non-responders. Though biomarkers have guided ICI precision therapy to some extent, they still only benefit a restricted number of patients (6). Computer models can simulate and reproduce dynamic changes in the immune system during immunotherapy, conferring unparalleled advantages in inferring the law of immunotherapy and predicting tumor ICI immunotherapy response (7, 8).

**Figure 1:**
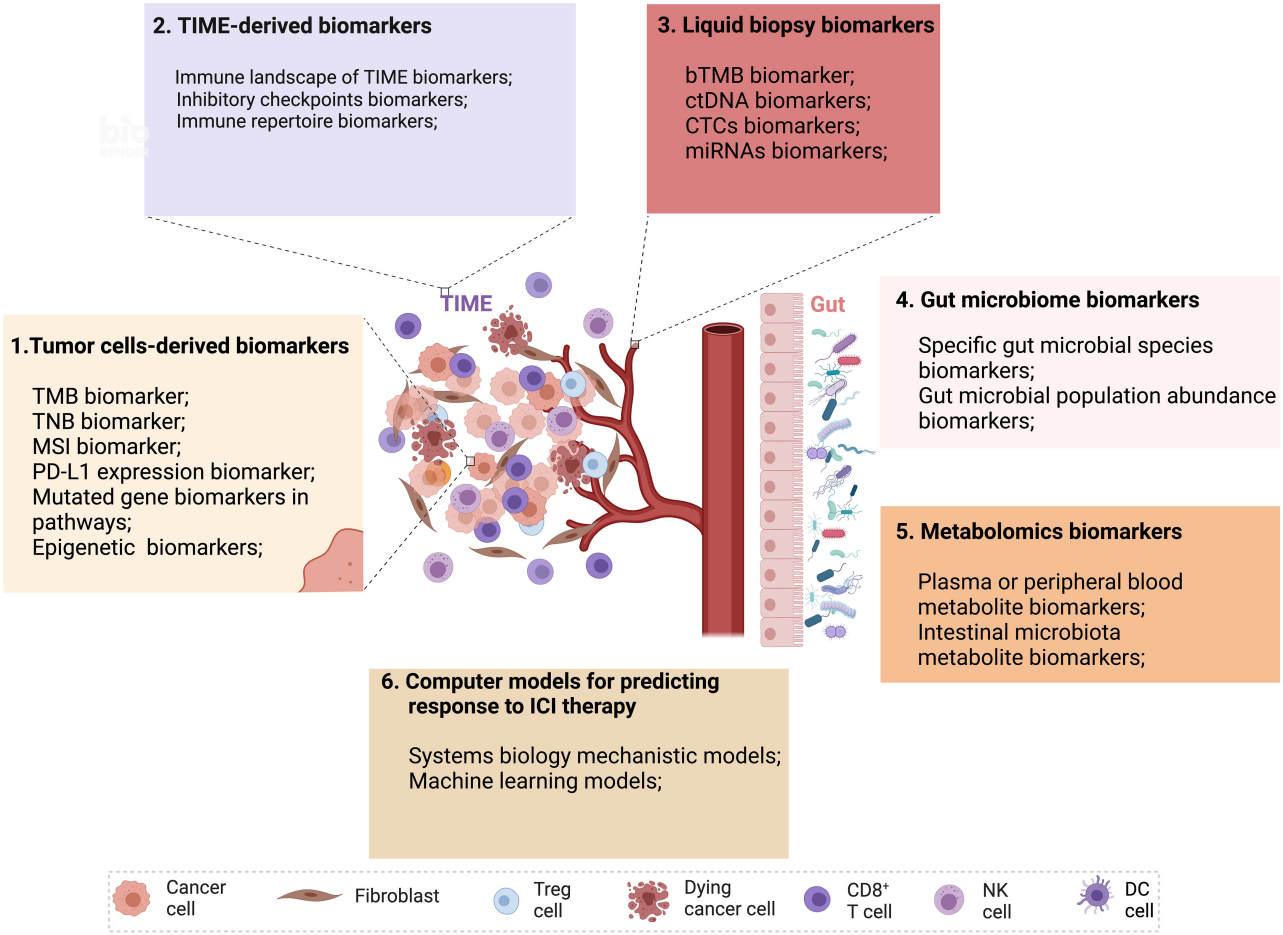
Multidimensional biomarkers and computer models to predict the response to ICI tumor immunotherapy. Multidimensional biomarkers include tumor cells-derived biomarkers and TIME-derived biomarkers. 1. Tumor cells-derived biomarkers include TMB, TNB, MSI, PD-L1 expression, mutated gene biomarkers in pathways, and epigenetic biomarkers. 2. TIME-derived biomarkers include the immune landscape of TIME biomarkers, inhibitory checkpoint biomarkers, and immune repertoire biomarkers. 3. Liquid biopsy biomarkers (bTMB, ctDNA, CTCs, miRNAs). 4. Gut microbiome biomarkers (Specific gut microbial signatures biomarkers, gut microbial population abundance biomarkers). 5. Metabolomics biomarkers (Plasma or peripheral blood metabolite biomarkers, intestinal microbiota metabolite biomarkers). 6. Computer models for predicting response to ICI therapy (Systems biology mechanistic models and machine learning models) (Created with Biorender). bTMB, blood-based tumor mutational burden; CTCs, circulating tumor cells; ctDNA, circulating tumor DNA; miRNAs, microRNAs; MSI, microsatellite instability; TIME, tumor immune microenvironment; TMB, tumor mutational burden; TNB, tumor neoantigen burden.

In this review, we focused on reviewing predictive biomarkers that impact the effectiveness of ICI therapy from the perspective of tumor cells, TIME, and other dimensions (**Figure 1**). We also discussed the various detection techniques for these biomarkers. Moreover, we summarized the detailed information of these biomarkers, outlining the datasets they adopt, their advantages and disadvantages, and the methods or indicators for evaluation (**Supplementary Table S1**). We also explored the limitations and challenges of biomarker research for ICI therapy, such as the impact of tumor heterogeneity, the lack of standardization in biomarker detection and analysis, and the difficulty in translating research findings into clinical practice.

Furthermore, we comprehensively reviewed computational models for predicting the response to ICI treatment, including mechanistic models based on bottom-up prior knowledge-based systems biology and top-down data-driven ML models and hybrids of both (**Figure 1**). We also summarized the details of these models, outlining the datasets they adopt, their advantages and disadvantages, and the methods or indicators for evaluation. A summary of validated biomarkers and computer models, along with their predictive performance and clinical utility, has been incorporated to assist readers in quickly identifying the most relevant biomarkers and models for their research or clinical practice.

## 2 Biomarkers in immune checkpoint inhibitor (ICI) Immunotherapy

### 2.1 Tumor cells-derived biomarkers

#### 2.1.1 Tumor mutational burden (TMB) biomarker

TMB measures the quantity of somatic gene-coding mutations, mainly consisting of single nucleotide variants (SNVs) and insertions and deletions (INDELs) per DNA megabase (Mb). TMB values positively correlate with the response to ICI therapy in various cancer types, including bladder cancer, non-small cell lung cancer (NSCLC), head and neck cancer and melanoma (9–12). However, a mere small proportion of mutations may translate into neoantigens that activate T cell responses (13). There are also other causes of the lack of efficacy of ICI in TMB high tumors, such as the immunosuppressive environment of the tumor (14), or poor infiltration of immune cells (15). Hence, TMB-high may not accurately predict the effectiveness of ICI therapy in all types of cancer (16), whereas some patients with lower TMB may still benefit from PD-1/PD-L1 blockade therapy (17). These cases show that TMB cannot accurately distinguish tumor patients who respond effectively to ICI therapy.

**Figure 2:**
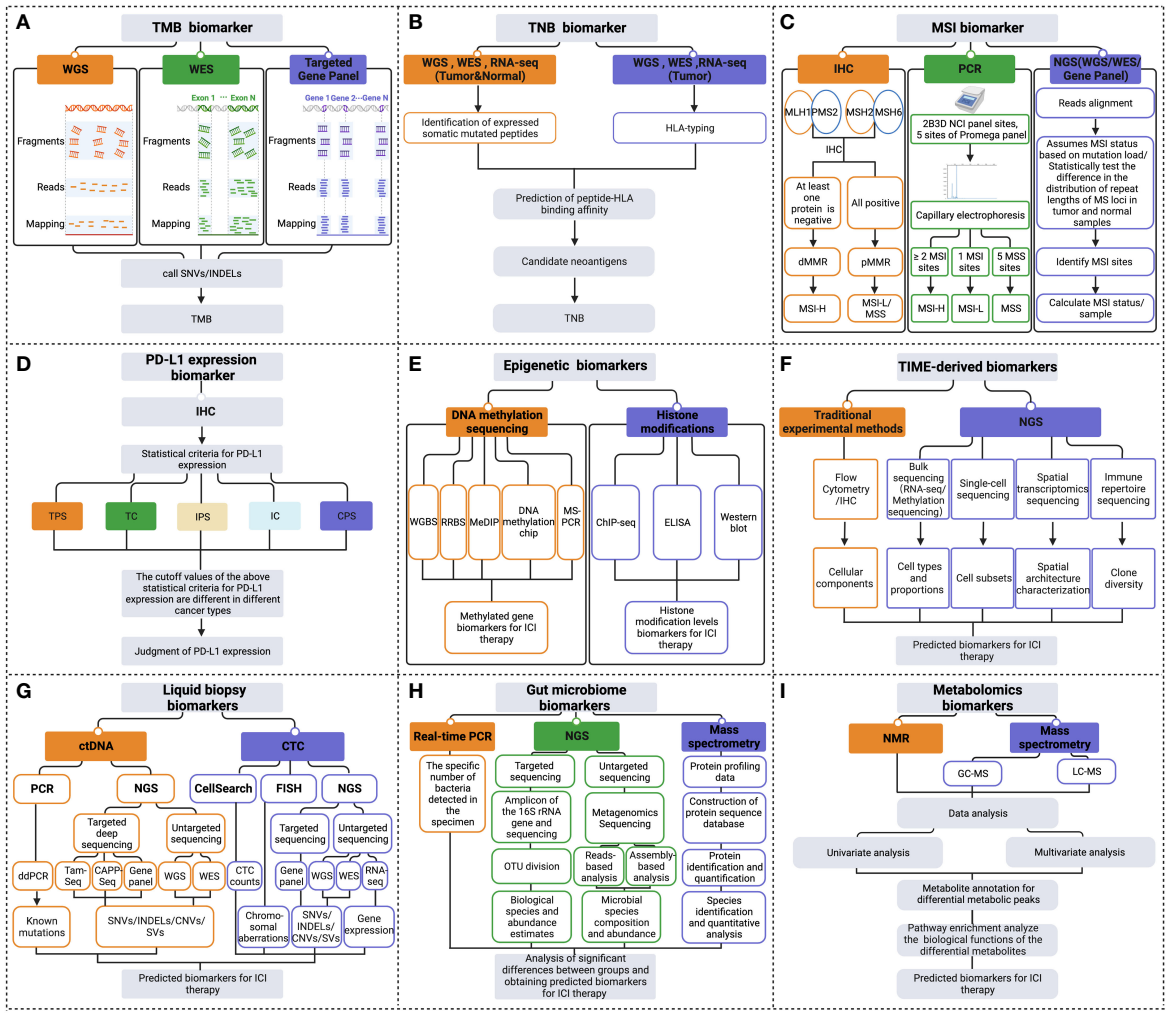
Multidimensional biomarkers detection methods to predict the response to ICI immunotherapy. The detection methods for tumor cells-derived biomarkers include **(A)** TMB biomarker detection methods; **(B)** TNB biomarker detection methods; **(C)** MSI biomarker detection methods; **(D)** PD-L1 expression biomarker detection methods; **(E)** Epigenetic biomarkers detection methods. **(F)** Detection methods of TIME-derived biomarkers. **(G)** Liquid biopsy biomarkers detection methods. **(H)** Gut microbiome biomarkers detection methods. **(I)** Metabolomics biomarkers detection methods (Created with Biorender). ChIP-seq, chromatin immunoprecipitation followed by sequencing; CNVs, copy number variations; CPS, combined positive score; ddPCR, droplet digital PCR; dMMR, deficient MMR; ELISA, enzyme-linked immunosorbent assay; FISH, fluorescence *in situ* hybridization; GC-MS, gas chromatography- mass spectrometry; HLA, human leukocyte antigen; IC, immune cell; IHC, immunohistochemistry; INDELs, insertions and deletions; IPS, immune proportion score; LC-MS, liquid chromatography- mass spectrometry; MeDIP, methylated DNA immunoprecipitation; MSI-H, MSI-high; MSI-L/MSS, MSI-low/microsatellite-stable; MS-PCR, methylation-specific polymerase chain reaction; NGS, next-generation sequencing; NMR, nuclear magnetic resonance; OUT, operational taxonomic units; PCR, polymerase chain reaction; pMMR, proficient MMR; RNA-seq, RNA-sequencing; RRBS, reduced representation bisulfite sequencing; SNVs, single nucleotide variant; SVs, structural variation; TC, tumor cell; TPS, tumor proportion score; WES, whole exome sequencing; WGBS, whole genome bisulfite sequencing; WGS, whole genome sequencing.

TMB detection technologies primarily consist of whole genome sequencing (WGS), whole exome sequencing (WES), and gene panels (**Figure 2A**). Although WGS offers a comprehensive view for TMB detection, its usage is restricted due to high costs, long processing times, and intensive data analysis needs. On the other hand, while WES is typically regarded as the most accurate method for TMB detection, it is often too costly and complex for routine clinical use. As TMB values calculated based on gene panels are generally considered reliable due to their consistency with TMB results measured from WES data, gene panel-based technologies are frequently employed in clinical practice (18). In practical applications, the cutoff value of TMB is influenced by multiple factors such as tumor types, sequencing platforms, number of genes detected, and algorithms, making it difficult to achieve consistency (19). And there is no clear optimal TMB value to maximize efficacy in the pan-tumor population. Nevertheless, the combination of TMB and other practical markers such as PD-L1 expression, microsatellite instability (MSI) status can optimize the stratification of responders and non-responders of patients receiving ICI therapy (20, 21).

#### 2.1.2 Tumor neoantigen burden (TNB) biomarker

Tumor neoantigens, as the primary target recognized by cytotoxic T cells, play a crucial role in the success of immunotherapy. The number of tumor neoantigens per megabase of the tumor genome is known as TNB (22, 23). In clinical trials of PD-1/PD-L1 antibody therapy, patients with a higher TNB had a more prolonged overall survival (OS) (24, 25). However, resistance to ICI treatment has also been reported in patients with high TNB (26), indicating the need for further optimization of TNB as a biomarker to predict ICI immunotherapy (27).

Many studies that evaluate TNB focus on predicting tumor neoantigens, which are traditionally assessed based on the prediction of binding affinity between the peptide and human leukocyte antigen (HLA) (28) (**Figure 2B**). However, this method has limited accuracy due to factors such as sequencing data quality, the accuracy of biological information tools, and the complexity of tumor genetics. Therefore TNB inferred from identified neoantigens does not have higher accuracy than TMB in predicting ICI immunotherapy (29). To improve the accuracy of neoantigen prediction, neoantigen heterogeneity should be considered, including truncal and subclonal mutations and changes in neoantigen immunogenicity resulting from immunoediting. Recent studies have developed novel algorithms such as the Cauchy-Schwarz index neoantigen (CSiN) score and the immune-editing-optimized tumor neoantigen load (ioTNL) algorithm to account for neoantigen heterogeneity and predict the response to ICI therapy in various cancers (30, 31). Additionally, multi-omics data, including tumor genomics, transcriptomics, epigenomics, and proteomics, can provide more molecular information for detecting neoantigen load and predicting immunotherapy efficacy (13, 32). Tumor genomics serves as the foundation for predicting neoantigen load (33), yet the integration of multi-omics data can further refine this prediction. For example, transcriptomic analysis can reveal which mutated genes are actually being expressed at the RNA level (34), thus identifying potential neoantigens that can be transcribed and translated into proteins, essential for recognition by the immune system. Epigenomic data, such as DNA methylation analysis, aids in understanding the regulatory mechanisms of gene expression, pinpointing which neoantigens might not be effectively presented due to epigenetic modifications (35). Proteomics, by directly measuring proteins on the tumor cell surface and inside, can confirm which neoantigen peptides are processed and presented by tumor cells to the immune system (36). The combined utilization of these multi-omics data not only enhances the accuracy of neoantigen load detection but also assists in more accurately predicting patients’ responses to immunotherapy.

#### 2.1.3 Microsatellite instability (MSI) biomarker

MSI is a reliable biomarker for predicting the effectiveness of ICI immunotherapy in clinical practice.MSI refers to INDELs mutations in repeating microsatellites units due to a functional defect in the mismatch repair (MMR) system that impairs the repair of DNA replication errors (37). Loss of MMR protein function caused by MMR gene mutations leads to MSI-high (MSI-H) (38). The food and drug administration (FDA) approved anti-PD1 blocking antibody pembrolizumab to be used in the therapy of unresectable or metastatic solid tumors in patients with MSI-H/deficient MMR (dMMR) in, 2017. This was the first time the FDA had approved an antitumor treatment based on biomarkers rather than tumor origin (39). However, MSI-H is relatively rare in most cancer types, except for endometrial and colorectal cancer, with a prevalence ranging from 0% to 16.5% across different cancer types (40). Although the available evidence suggests that MSI-H/dMMR is an effective biomarker to predict the effectiveness of ICI immunotherapy in specific cancer types, more comprehensive data is necessary to confirm the overall value of this biomarker.

Various MSI assays have been developed, such as immunohistochemistry (IHC), multiplex fluorescent polymerase chain reaction (PCR) capillary electrophoresis, and next-generation sequencing (NGS) (41) (**Figure 2C**). The IHC method primarily detects the expression of MMR proteins in the nucleus, including MutL homologue 1 (MLH1), postmeiotic segregation 2 (PMS2), MutS homologue 2 (MSH2), and MutS homologue 6 (MSH6). If any of these MMR proteins are absent, it is considered dMMR, which is thought to cause high MSI. Conversely, when all four MMR proteins are expressed, it is considered low MSI or microsatellite-stable (MSS), indicating proficient MMR (42, 43). PCR detection of MSI mainly adopts the method of multiplex fluorescent PCR combined with capillary electrophoresis (44). The detection sites used to determine MSI status include the 2B3D National Cancer Institute (NCI) panel sites (including two mononucleotide repeat unit sites and three dinucleotide repeat unit sites) and five mononucleotide repeat unit sites of the Promega panel (45, 46). NGS commonly detects MSI, including WGS, WES, or gene panel (47). NGS detection of MSI includes two main methods. The first method assumes MSI status based on mutation load. The second method measures the status of MSI by statistically examining differences in the distribution of read numbers of a set of microsatellite loci with different repeat lengths in tumor and standard samples (48). Overall, the various assays available for detecting MSI status in tumors offer clinicians a range of options. Therefore, it is essential to evaluate and choose the appropriate method for clinical use carefully.

#### 2.1.4 PD-L1 expression biomarker

PD-L1 expression is the first clinically validated biomarker to predict the curative effectiveness of PD-1/PD-L1 antibodies (49). However, there are certain limitations to its accuracy. One retrospective clinical study revealed that just 28.9% of patients could be predicted for ICI treatment efficacy using PD-L1 expression as a useful biomarker (50). Furthermore, in recent studies, treatment with PD-1/PD-L1 blockade is still effective for some patients with negative PD-L1 expression (51, 52). The efficacy of PD-L1 as a reliable biomarker may be influenced by the heterogeneity of tumors and the tumor microenvironment (TME). The TME is defined as the complex milieu surrounding a tumor, which includes a variety of cell types, blood vessels, immune cells, and extracellular components. This definition also takes into account the dynamic nature of patients’ immune responses, highlighting the intricate interactions that could affect biomarker reliability (53, 54).

PD-L1 expression is mainly detected in clinical settings using IHC methods. PD-L1 expression status can be assessed by measuring various scoring metrics, including tumor proportion score (TPS), tumor cell (TC) score, immune proportion score (IPS), immune cell (IC) score, as well as combined positive score (CPS) (55–57). These scores are methods to quantify the level of PD-L1 expression on tumor and immune cells. The statistical thresholds for PD-L1 expression assessment vary across different cancer types (**Figure 2D**). Establishing a standardized benchmark for expression assessment is necessary to improve the accuracy of using PD-L1 expression as a predictor in guiding ICI treatment.

#### 2.1.5 Mutated gene biomarkers in pathways

**Figure 3:**
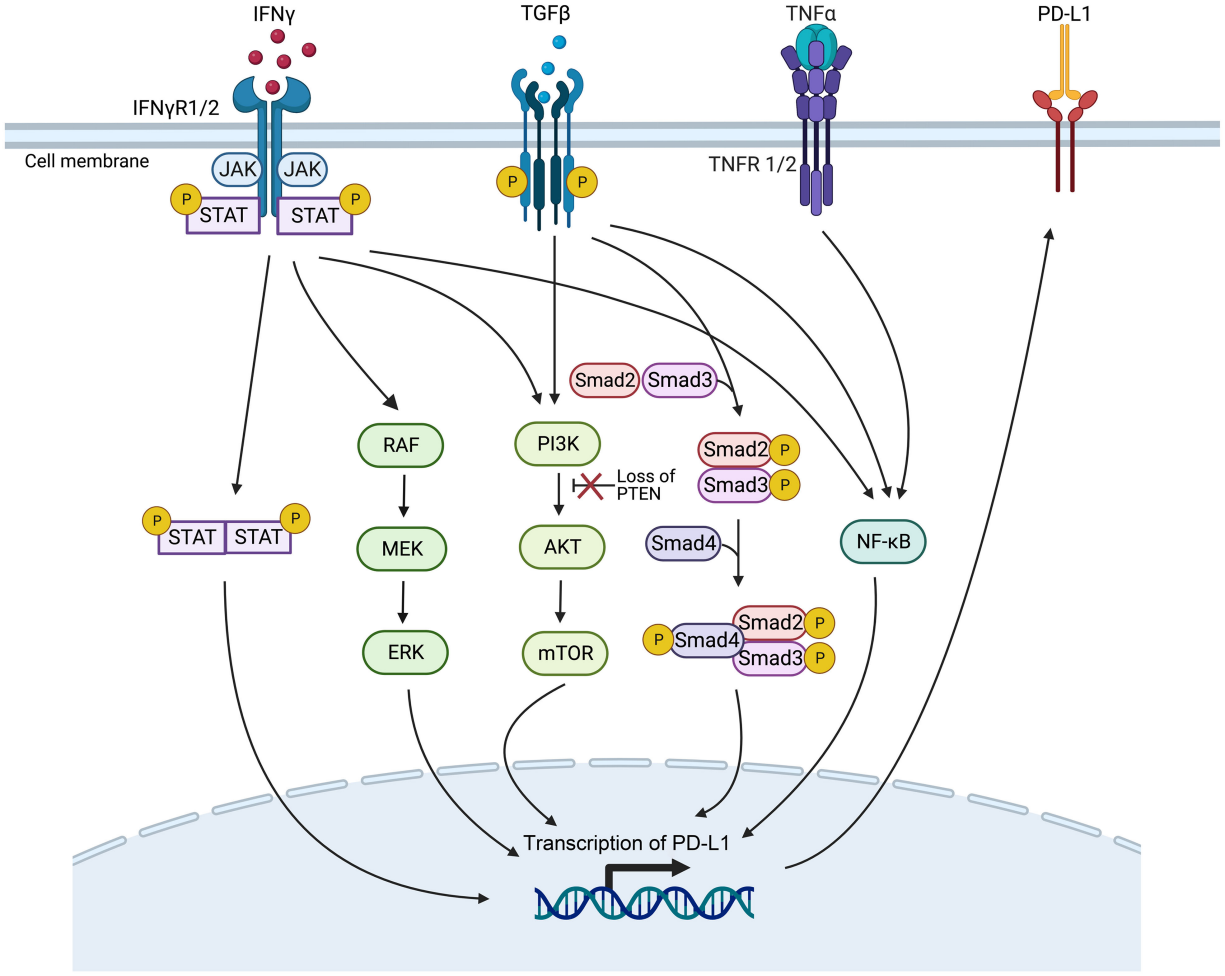
Genetic mutations or gene expression changes in various signaling pathways affect PD-L1 expression. The signaling ways include IFN-γ, MAPK, PI3K, TGF-β, and the TNF-α signaling pathways (Created with Biorender). JAK/STAT, Janus kinase/signal transducers and activators of transcription; mTOR, mammalian target of rapamycin; NF-κB, nuclear factor κB; PTEN, phosphatase with tensin homology.

Oncogenic mutations or gene expression changes in cancer cells can activate or inhibit various signaling pathways, such as the interferon-γ (IFN-γ), mitogen-activated protein kinase (MAPK), phosphatidylinositol 3-kinase (PI3K), transforming growth factor-β (TGF-β), and tumor necrosis factor-α (TNF-α) pathways, which can affect PD-L1 expression (**Figure 3**) and the TME, ultimately impacting ICI therapy efficacy. These pathway-related genetic mutations or gene expression changes may be potential biomarkers for predicting ICI immunotherapy efficacy (58). These biomarkers are presently subject to clinical and preclinical investigations. To date, they have not received approval from the FDA for clinical application (59).

The IFN-γ pathway is activated when IFN-γ receptor 1/2 (IFNγR1/2) on tumor cells are recognized by IFN-γ, activating the Janus kinase/signal transducers and activators of transcription (JAK/STAT) pathway, then inducing the expression of PD-L1 to inhibit the ICI therapy (60). Gene mutations in proteins related to the IFN-γ pathway, such as IFNγR1/2 and signal transducer and activator of transcription 1 (STAT1), can lead to resistance to ICI therapy, making them potential predictive biomarkers of efficacy (61, 62).

Gene mutations that activate or inhibit the MAPK pathway may also serve as practical biomarkers to predict PD-1/PD-L1 blockade therapy. In lung adenocarcinoma, growth factors and IFN-γ can up-regulate PD-L1 expression levels through MAPK signaling and affect response to immunotherapy (63). In addition, inhibition of the mitogen-activated protein kinase kinase (MEK) and B-Raf proto-oncogene, serine/threonine kinase (BRAF) mutations can enhance the curative efficacy of PD-1/PD-L1 inhibitors (64).

Deletion of phosphatase and tensin homolog (PTEN), a tumor suppressor gene, in tumor cells causes the serine/threonine kinase Akt phosphorylation, enhancing the PI3K/Akt signaling pathway and ultimately up-regulating the expression of PD-L1, leading to T cell inactivation (65). PTEN-deficient metastatic melanoma patients have lower levels of inflammation-related gene expression and tumor-infiltrating lymphocytes, reducing anti-PD-1 therapy response (66).

High expression levels of TGF-β ligand TGFB1 and its receptor TGFBR2, two crucial genes of the TGF-β signaling pathway, are associated with non-response and decreased OS in tumor patients treated with ICI immunotherapy (67). It has been demonstrated that TGF-β can boost the expression of PD-L1 in a Smad2-dependent manner (68). TGF-β can activate the PI3K/Akt/mammalian target of rapamycin (mTOR) and Smad3 pathways to induce epithelial-mesenchymal transition (EMT) (69), which has been associated with the up-regulated expression of PD-L1 (70). Furthermore, TGF-β up-regulates the presentation of the myocardin related transcription factor-A (MRTF-A), promoting the combination of nuclear factor kappa-light-chain-enhancer of activated B cells/p65 subunit (NF-κB/p65) and the PD-L1 promoter, ultimately leading to the activation of PD-L1 expression (71). In addition, TNF-α can activate the NF-κB pathway, leading to the demethylation of the cluster of differentiation 274 (CD274) promoter and the promotion of PD-L1 expression (72). However, high levels of PD-L1 expression after ICI treatment have been associated with poorer treatment outcomes (73).

In the identification of mutated gene biomarkers within signaling pathways, a suite of advanced detection technologies are currently employed, with NGS playing a pivotal role. NGS facilitates whole-genome sequencing, whole-exome sequencing, and targeted gene panel sequencing, not only identifying known genetic mutations but also uncovering novel mutation events, thereby significantly expanding the scope of mutated gene biomarker detection (74). Additionally, quantitative real-time PCR (qPCR) and digital PCR (dPCR), renowned for their exceptional sensitivity and rapid response, have become the methods of choice in both clinical and research settings for detecting specific known mutations. fluorescence *in situ* hybridization (FISH) and microarray technologies also provide precise capabilities for mutation gene localization and expression level analysis. With the continuous advancements in biotechnology, emerging techniques such as single-cell sequencing and RNA sequencing are being employed in mutation detection, offering more accurate tools for cellular-level variant analysis (75–77). The integrated application of these advanced technologies, coupled with robust bioinformatics analysis, now enables us to more comprehensively and deeply unravel mutated genes within signaling pathways, thereby enhancing the precision in detecting predictive biomarkers for cancer ICI immunotherapy efficacy.

#### 2.1.6 Epigenetic biomarkers

Epigenetic variation in tumor cells has been linked to tumor progression and changes in immune response, suggesting that it can serve as a valuable biomarker for predicting the effectiveness of ICI therapy (78). This variation includes DNA methylation, modifications of histones, and other chromatin remodeling activities (79). Guerreiro et al. investigated that the combination of DNA repair gene *RAD51B* promoter methylation *RAD51B^me+^* with PD-L1^+^ was more effective in predicting the response to ICI therapy than either marker alone in NSCLC (80). Darvin et al. found that histone acetylase overexpression is involved in the expression of PD-L1 induced by EMT, contributing to immune evasion (81). Loss of function in polybromo 1 (PBRM1), a subunit of the switch/sucrose‐non‐fermentable (SWI/SNF) chromatin remodeling complex, is related to improved clinical outcomes in patients receiving PD-1 antibody therapy (82).

Recent advancements in DNA methylation sequencing technology have enabled the identification of differentially methylated genes or methylation signatures as predictive biomarkers of the efficacy of ICI treatment (83). This technology includes whole genome bisulfite sequencing (WGBS), reduced representation bisulfite sequencing (RRBS), methylated DNA immunoprecipitation (MeDIP), DNA methylation chips, and methylation-specific polymerase chain reaction (MS-PCR), among others (**Figure 2E**). MS-PCR is commonly used in clinical testing to analyze site-specific methylation of individual CpG islands (84). Chromatin immunoprecipitation followed by sequencing (ChIP-seq) is usually used to investigate histone-specific modifications at specific locations throughout the genome (85). Additionally, the overall levels of histone modifications can be assessed through enzyme-linked immunosorbent assay (ELISA) or western blot techniques (86) (**Figure 2E**).

### 2.2 Tumor immune microenvironment (TIME)-derived biomarkers

#### 2.2.1 Immune landscape of TIME biomarkers

Understanding the immune landscape of the TIME is critical in predicting the efficacy of immunotherapy, as an immunosuppressive TIME can hinder its effectiveness. Cell type, abundance, location, and function within the TIME may impact tumor immunotherapy efficacy (58). A range of methods is available to quantify the different cellular components of tumor tissue. Traditional experimental techniques, such as flow cytometry and IHC, may have limited utility due to low tissue availability or high costs (87). NGS data for analyzing immune cells primarily come from bulk sequencing, such as transcriptome and methylation data, single-cell sequencing data, and spatial transcriptome sequencing data (**Figure 2F**). The transcriptome and methylation data analysis can be divided into three methods: a non-reference strategy and two reference-based methods - deconvolution and scoring (88). More recently, single-cell sequencing has enabled the characterization of immune cell status within the TIME at a single-cell level, allowing for identifying novel tumor-associated immune cell subsets at a finer resolution (89). The development of spatial transcriptomics sequencing has allowed the study of spatial distribution characteristics within the TIME, thereby obtaining spatial biomarkers to predict the effect of immunotherapy (90). In the tumor microenvironment (TME), fibrosis, marked by an excessive buildup of the extracellular matrix, particularly collagen, leads to hardened and thickened tissue structures. This condition can hinder immune cell infiltration and functionality, thereby impacting the efficacy of ICI therapy. As such, fibrosis within the TME is identified as a significant biomarker, aiding in the prediction of ICI therapeutic outcomes. The influence of fibrosis on both the TME structure and immune cell functions within the TIME highlights its critical role in modulating tumor immune responses, underscoring the importance of further investigation into fibrosis for enhancing immunotherapy strategies (91, 92).

#### 2.2.2 Inhibitory checkpoints biomarkers

In addition to CTLA-4 and PD-1/PD-L1, the TIME contains other inhibitory checkpoints such as T-cell immunoglobulin and mucin-domain-containing molecule 3 (TIM-3) plus TIM-3 ligands, lymphocyte-activation gene 3 (LAG-3). TIM-3 up-regulated by T cells has also been proposed as a candidate biomarker to track the progress of ICI therapy. During treatment with PD-1/PD-L1 inhibitors, TIM-3 upregulation may suppress T helper cell type 1 (Th1) cell responses and decrease the expression of cytokines like TNF-α and IFN-γ, leading to drug resistance in cancer patients (61, 93). In addition, LAG-3 has been shown to up-regulate Treg activity and inhibit Teff activity, which can create a TIME that suppresses immune responses (94). Therefore, monitoring the expression of LAG-3 is crucial for predicting ICI therapy response, but the expression of LAG-3 is not routinely used in the clinic (59).

#### 2.2.3 Immune repertoire biomarkers

The immune repertoire consists of all the T cell receptors (TCRs) and B cell receptors (BCRs) in an individual. These receptors bind to self-antigens or foreign antigens, such as external viruses and bacteria, triggering an immune response. Therefore, TCRs and BCRs are crucial in immunotherapy (95, 96). For example, patients receiving anti-CTLA-4 therapy demonstrate longer survival when exhibiting low baseline TCR clonality and the presence of clones with large expansion after treatment. In contrast, these factors do not exist in patients receiving anti-PD-1 therapy (97). The finding suggests that TCR clones may be biomarkers for predicting the response to ICI therapy.

The clonal diversity of the immune repertoire is assessed by analyzing the sequence characteristics of the V(D)J recombination fragments of the complementarity-determining region 3 (CDR3) region from the receptor gene, which can be used as a biomarker to predict the therapeutic effect of ICI (**Figure 2F**). Early tools for sequencing analysis of immune repertoires include IgBLAST (98), iHMMune-align (99), and IMGT/V-QUEST (100). MiXCR can correct PCR errors and precisely analyze sequencing data for BCR and TCR (101). IGoR can accurately identify V(D)J rearrangements (102). TRUST4 finds many CDR3s with high precision and sensitivity (103). Integrating artificial intelligence algorithms with BCR and TCR sequencing data may lead to more accurate identification of patient subgroups likely to respond positively to ICI therapy.

### 2.3 Liquid biopsy biomarkers

In the past decade, the development of non-invasive techniques for tumor diagnosis, such as liquid biopsy, has dramatically accelerated the pace of tumor research (104). Potential sources for the development of fluid biopsy biomarkers for tumor ICI therapy include blood-based TMB (bTMB), circulating tumor DNA (ctDNA), circulating tumor cells (CTCs), and microRNAs (miRNAs), among others (105). As a non-invasive and replicable biomarker for forecasting ICI treatment efficacy, bTMB offers tremendous advantages in predicting clinical outcomes for cancer patients (106). Identifying alterations in ctDNA levels can predict a patient’s response to immunotherapy sooner than traditional imaging evaluations (107). In addition, positive PD-L1 expression status in CTCs can expect a response to immunotherapy (108). Incorvaia et al. reported that directly identified miRNA expression profiles of patients before and after treatment could be used to assess the dynamic molecular changes underlying PD-1 inhibitor nivolumab treatment and predict the response to treatment (109).

Detection of ctDNA in liquid biopsy includes quantitative PCR and NGS (110) (**Figure 2G**). Droplet digital PCR (ddPCR) is an advanced form of quantitative PCR and provides exceptional sensitivity and precision for nucleic acid quantification (111). NGS detects ctDNA mutations using non-targeted sequencing and targeted deep sequencing methods. Non-targeted sequencing encompasses WGS and WES. Leary et al. used the PARE method to detect ctDNA genomic rearrangement breakpoints in plasma samples via whole genome analysis (112). Targeted deep sequencing, such as tagged-amplicon deep sequencing (Tam-Seq), cancer personalized profiling by deep sequencing (CAPP-Seq), and gene panel methods, captures specific genomic regions enriched in ctDNA by PCR or hybridization (113). NGS can identify ctDNA mutations, such as SNVs, INDELs, copy number variations (CNVs), structural variations (SVs), etc. Nevertheless, the sensitivity and specificity of ctDNA detection by NGS depend on sequencing depth, bioinformatic analysis pipelines, and parameters (113, 114). CTC enrichment and detection can be achieved through biophysical and antibodies-based methods. Antibody-based methods detect cell surface markers, such as epithelial cell adhesion molecule (EpCAM) on CTCs. For example, the CellSearch^®^ system, an FDA-approved automated CTC counting system, uses EpCAM-based detection (115). FISH can also detect CTCs, where chromosomal aberrations can be observed (116). NGS detects CTCs by non-targeted sequencing (WGS, WES, RNA-sequencing (RNA-seq)) and targeted sequencing gene panel methods. WGS, WES, and gene panels can detect CTC mutations, including SNVs, INDELs, CNVs, SVs, etc. (117–120), and RNA-seq allows for gene expression detection (121). Sequencing techniques enable the identification of differentially mutated or expressed genes in ICI treatment-responsive and non-responsive patients, making liquid biopsies a valuable source of biomarkers for ICI therapy (**Figure 2G**).

### 2.4 Gut microbiome biomarkers

Regulation of the intestinal flora on human immunity directly affects the effect of tumor immunotherapy (122–124). The impact of PD-1/PD-L1 blocking treatment is compromised in antibiotic-experienced cancer patients, and survival is significantly reduced (125–127). Melanoma patients exhibited distinct microbial signatures in their intestines, with *Lachnospiraceae* spp. being associated with a favorable clinical response, while *Streptococcaceae* spp. was associated with an unfavorable clinical response (128). Zheng et al. found that patients with good responses to treatment had a higher population richness of the gut microbiota and gene counts than cancer patients with poor answers. The compositional richness of the intestinal microbiota is closely related to the effect of immunotherapy (129). Therefore, the gut microbiota can affect and predict the clinical impact of immunotherapy on cancer.

Various molecular detection methods are available for analyzing intestinal microbes, including quantitative real-time PCR, targeted NGS such as 16S ribosomal RNA (rRNA) gene amplicon analysis, non-targeted NGS sequencing, and mass spectrometry (MS) (130) (**Figure 2H**). Quantitative fluorescence PCR in real-time is a simple and effective method for detecting the specific number of bacteria in the sample, with strong specificity (131). The data analysis of 16S amplicon sequencing includes operational taxonomic units (OTUs) classification and estimation of biological species and abundance, enabling significant difference analysis between the response and non-response groups to obtain biomarkers for efficacy prediction of ICI treatment (132, 133). Metagenomics extracts the total DNA of environmental microorganisms for non-targeted sequencing (134). Two analysis methods are commonly used for metagenomic sequencing to obtain the composition and abundance of microbial species. The first method directly compares the reads to reference genomes or feature gene sets. The second method involves getting high-quality bacterial genomes based on assembly and then using genome sequence annotation and alignment to obtain species composition and abundance. Combining these two methods usually yields more accurate results (135). MS technology is used for metaproteomics detection. It can accurately and efficiently identify the distinct protein components in human gut microbiota, enabling species identification and quantitative analysis (136, 137). Predictive biomarkers of ICI therapy can be identified by analyzing the significant differences in gut microbiome species or abundance between patients who respond to ICI treatment and those who do not.

### 2.5 Metabolomics biomarkers

Metabolites are crucial in exploring new predictive biomarkers for ICI therapy in cancer patients (138). In one investigation, the analysis of metabolites in plasma from NSCLC patients before and after nivolumab treatment using liquid chromatography-MS (LC-MS) or gas chromatography-MS (GC-MS) showed that a combination of four metabolites could effectively predict the therapeutic effect of the PD-1 inhibitor (139). Higher cholesterol levels in the bloodstream have been linked to longer OS and progression-free survival (PFS) in cancer patients treated with ICI (140). Short-chain fatty acids (SCFAs), metabolites from the host intestinal microbiota, are critical in regulating immune cell response. SCFAs-focused microbial metabolites may be a new biomarker to predict the immunotherapy response (141).

Nuclear magnetic resonance (NMR) spectroscopy and MS are widely used techniques for directly detecting metabolites in metabolomics research (142, 143). MS is divided into LC-MS and GC-MS (144) (**Figure 2I**). The spectrum produced by NMR is linearly related to the compound concentration. However, due to its low sensitivity, NMR is often used to identify and analyze simple or purified samples. On the other hand, combining MS and chromatographic separation offers high sensitivity and specificity. The MS raw data analysis tools include XCMS (145), MZmine (146), and IP4M (147). Statistical analysis, including univariate and multivariate analysis, is primarily used to identify differential metabolite peaks between responders and non-responders to ICI therapy (148). Then, the annotation of metabolites is performed on the differential metabolic peaks, and the biological functions of the differential metabolites are analyzed through pathway enrichment, which is used to predict the therapeutic effect of ICI.

### 2.6 Challenges, limitations, and future perspectives

It’s essential to acknowledge the challenges and limitations of this field, such as the impact of tumor heterogeneity, the lack of standardization in biomarker detection, and the complexities of translating research into clinical practice. Further research into these areas will likely yield more nuanced insights and refinements in biomarker discovery and utilization. At the same time, we are confronted with the dual challenge of integrating diverse omics data without substantially increasing costs and identifying the minimal biomarker combination that can accurately predict responses to immunotherapy.

Tumor heterogeneity serves as a principal source of variability in treatment responses, a phenomenon not only observed between distinct tumors but also within individual tumors. This directly impacts the expression and detection of biomarkers, thereby influencing the prediction of treatment outcomes. Future research should delve deeper into the essence of tumor heterogeneity and explore strategies for overcoming this challenge through precise biomarker combinations.

Moreover, the lack of standardization in biomarker detection warrants attention. Variabilities in detection methodologies and standards across different laboratories compromise the comparability of results, adding layers of complexity to the translation of research findings into clinical practice. Thus, establishing unified detection standards and methodologies is imperative for enhancing the accuracy and reliability of biomarker detection.

The complexity of translating research outcomes into clinical applications is equally critical. Despite the potential of research to provide valuable biomarker insights, applying these discoveries in a clinical setting necessitates overcoming several hurdles, including cost, feasibility of technology transfer, and applicability across diverse patient populations.

Looking forward, the integration of various omics data, such as genomics, transcriptomics, and metabolomics, promises to offer a more comprehensive view of the tumor and its microenvironment. Although such integrative research requires sophisticated modeling techniques to process and analyze extensive datasets, it also poses significant cost challenges. Therefore, balancing the enhancement of predictive accuracy with cost control emerges as a pressing issue that demands resolution.

Identifying the minimal combination of biomarkers for effective prediction of immunotherapy responses is both complex and crucial. This task involves not only assessing the dependency of biomarkers on the tumor’s histological type but also considering the use of advanced technologies like machine learning and artificial intelligence to discern the most promising biomarker combinations from data. Strategies to address tumor heterogeneity might include the development of new biomarkers that reflect various aspects of the tumor, such as gene expression, metabolic state, and immune environment. Additionally, customizing treatment plans based on specific tumor characteristics of patients could improve therapeutic effectiveness and precision.

Overall, future research requires not only technological and methodological innovation but also conceptual breakthroughs to tackle the current challenges and limitations. By integrating and analyzing multiple types of omics data, developing novel biomarkers, and formulating precise treatment strategies, we can advance toward more accurately predicting responses to immunotherapy. Interdisciplinary collaboration, involving the concerted efforts of biologists, data scientists, clinicians, and patients, will be key to achieving this goal. Through such collaboration, we anticipate overcoming existing challenges and providing patients with more effective and personalized treatment options.

## 3 Computer models to predict the response to ICI therapy

**Figure 4:**
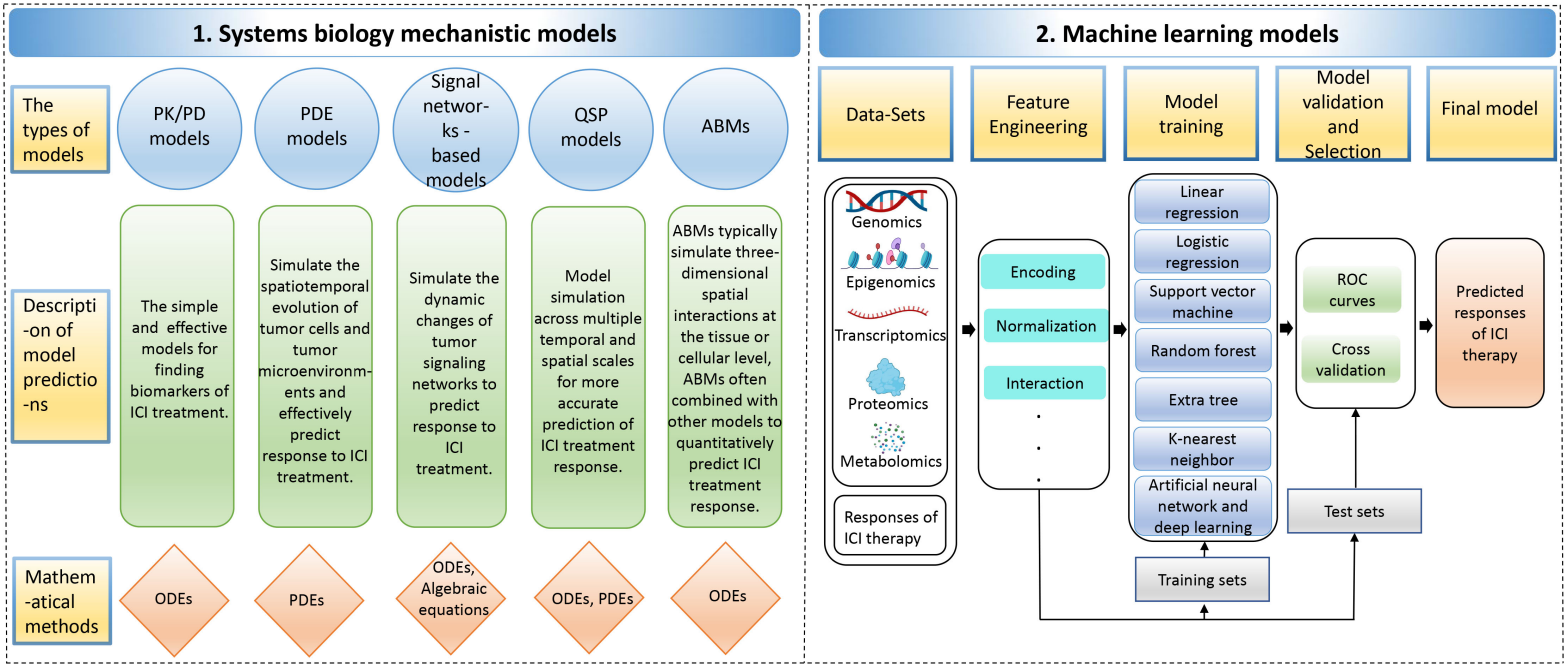
The computer models for predicting responses to immune checkpoint inhibitor therapy. The computer models include 1. Systems biology mechanistic models; 2. Machine learning models. ABMs, agent-based models; ODEs, ordinary differential equations; PDEs, partial differential equations; PK/PD, pharmacokinetic/pharmacodynamic; QSP, quantitative systems pharmacology; ROC, receiver operating characteristic.

While the administration of ICI antibodies via intravenous infusion is a straightforward process, the real complexity in tumor immunotherapy lies in understanding the biological mechanisms of the treatment and accurately predicting patient responses. This challenge is exacerbated by the intricate interplay of immunosuppressive and immunostimulatory components within the tumor microenvironment, which significantly complicates the prediction of therapeutic outcomes (149, 150). A system-level framework is necessary to comprehend the dynamic temporal and spatial relationships between cellular and molecular types. Mechanistic modeling in systems biology can help achieve this by modeling multivariate biomarkers with dynamic characteristics and extracting essential parameters based on prior knowledge, allowing for systematic evaluation of immunotherapy predictive biomarkers (151). Various mechanistic models, including pharmacokinetic/pharmacodynamic (PK/PD) models, partial differential equations (PDE) models, signal network-based models, quantitative systems pharmacology (QSP) models, and agent-based models (ABMs), can be used for predicting the efficacy of ICI therapy. **Supplementary Table S2** describes the detailed information of these mechanistic models, including the advantages and disadvantages of the models, the datasets used, evaluation methods/metrics, biomarkers, etc. Data-driven ML models capture rules from complex cancer changes, resulting in good prediction results even without a complete understanding of the underlying biological mechanisms. Furthermore, the features and parameters of ML models contain a wealth of latent information that can be utilized to explore new biomarkers (152, 153) (**Figure 4**). Additionally, Hybrid models, by combining prior knowledge with multimodal data using computer models, help to discover more accurate biomarkers of systematic dynamics.

### 3.1 Knowledge-based mechanistic models

#### 3.1.1 Pharmacokinetic/pharmacodynamic (PK/PD) Models

PK/PD models are typically created using ordinary differential equations (ODEs), which involve abstracting data into dynamic systems (154). PK/PD models can be used to identify biomarkers for predicting the curative effects of anti-PD-1/PD-L1 (7). For instance, Netterberg et al. developed a PK/PD quantitative model to explore circulating biomarkers capable of predicting early tumor response to PD-L1 inhibitor atezolizumab in NSCLC (155). In addition, Liu et al. developed population pharmacokinetic (PPK) models to describe the PK characteristics of the PD-1 antibody nivolumab. It has been reported that the change in clearance rate of nivolumab is associated with disease state after treatment. Specifically, patients with better disease response and survival showed greater reductions in baseline clearance, leading to higher exposure to drugs during stable conditions (156).

#### 3.1.2 Partial differential equations (PDE) models

In addition to using ODEs, partial differential equations (PDEs) can also be applied to simulate spatiotemporal tumor growth and predict the effectiveness of ICI therapy (157). For example, Siewe et al. utilized the PDE system to create a mathematical model of cancer cells, immune cells, and cytokines to explain the primary drug resistance of PD-1 antibody therapy. This model also identified two cancer parameters that could potentially be biomarkers to predict the effectiveness of combination therapy using both anti-PD-1 and anti-TGF-β treatments (158).

#### 3.1.3 Signal networks-based models

The dynamics of tumor signaling networks in cancer patients treated with ICIs can be modeled using ODEs, which predict their response to immunotherapy. For instance, Brogden et al. used ODEs to model protein-protein interactions at each step in the signaling pathway and modeled the reaction at each specific node as the Michaelis-Menten equation. They constructed a patient-specific predictive calculation model based on annotating a patient-specific spectrum of genetically deleterious mutations in cancer networks. This model was utilized to predict the expression of various chemotactic and immunosuppressive factors, which can help predict the effectiveness of PD-1 blockade therapy in tumor patients (159).

#### 3.1.4 Quantitative systems pharmacology (QSP) models

Compared to clinical trials, QSP models have advantages in exploring biomarkers that can predict the response of PD-1/PD-L1 blocked therapy by spanning multiple temporal and spatial scales (160). Zhang et al. developed a personalized prediction model for the curative response to ICI in triple-negative breast cancer by integrating single-cell tumor data and a spatial QSP model. Their model, consisting of a four-compartment QSP model representing the entire patient and an ABM that simulated spatiotemporal cellular and molecular interactions within the tumor compartment’s three-dimensional space, accounted for antigen burden and TME heterogeneity (161).

#### 3.1.5 Agent-based models (ABMs)

ABMs are commonly used to simulate three-dimensional spatial interactions at the tissue or cell level and have been widely used to evaluate cancer drug resistance and efficacy (162). ABM combines other models to build multiscale agent models to simulate ICI treatment and achieve quantitative prediction of combinations of therapeutic biomarkers (163). Storey et al. constructed a multiscale model that combined ABM and PDE models to simulate the response of glioblastoma to combined treatment with PD-1 inhibitor therapy and oncolytic virus therapy. According to the model simulations, the level of tumor antigenicity, which is determined by the tumor-mediated proliferation rate of T cells, has a more significant impact on therapeutic outcomes than the killing efficiency of T cells (164). These studies emphasize the significance and practical value of using ABMs in conjunction with other models for deeply understanding and predicting cancer treatment responses, particularly in the context of complex dynamics at the tissue and cellular levels.

### 3.2 Machine learning (ML) models

ML approaches can extract multi-dimensional latent features from biological multi-omics sequencing data, including genomic, epigenomic, transcriptomic, proteomic, and metabolomic data, to explore predictive markers of efficacy in ICI therapy (165, 166). These ML approaches generally entail data collection and selection, feature engineering, model building, validation, optimization, and evaluation. Various ML algorithms have been employed in predicting the effectiveness of cancer immunotherapy, including linear regression, logistic regression, support vector machine (SVM), random forest, extra tree, k-nearest neighbor (KNN), artificial neural network (ANN) and deep learning, among others (167). In addition, hybrid models of systems biology and ML are explained (**Supplementary Table S3**).

#### 3.2.1 Linear regression models

A linear regression model can capture the potential linear relationship between ICI treatment response levels and tumor patients’ cell molecular profiles (168). Common linear regression models include the ridge regression model, the least absolute shrinkage and selection operator (lasso) regression model, and the elastic net regression model. Elastic net regression can learn a sparse model similar to lasso regression but also has the stability of ridge regression (169). Xue et al. initially identified CpG probes linked to the objective response rate (ORR) of PD-1/PD-L1 blockade therapy, and then developed a lasso regression model using these probes for predicting the treatment’s efficacy (170). Sun et al. created an elastic net regularized regression method to obtain a radiomic signature-based non-invasive predictor of tumor-infiltrating CD8 cells to predict the curative response of PD-1/PD-L1 antibodies (171).

#### 3.2.2 Logistic regression models

Compared to the linear regression model, the logistic regression model introduces nonlinear factors by introducing the signature mapping function. It estimates model parameters using the maximum likelihood method and gradient descent for classification tasks (172–174). For instance, Park et al. developed a multivariate logistic regression model to predict ICI treatment response in metastatic urothelial carcinoma, with high-risk groups predicted by the model associated with worse survival outcomes (175). Refae et al. utilized elastic net penalty logistic regression to predict the therapeutic effect of anti-PD-1/anti-PD-L1 based on single nucleotide polymorphisms (SNPs) data related to immunogenetics in advanced cancer patients. They identified gene SNPs related to TME associated with ICI treatment efficacy (176). Zhang et al. used lasso logistic regression to select dynamic serum markers for metastatic or recurrent nasopharyngeal carcinoma and developed a risk score prediction model for PD-1 inhibitor therapy. The model’s predicted probability of efficacy was consistent with the observed probability of prediction (177).

#### 3.2.3 Support vector machine (SVM)/random forest/extra tree/k-nearest neighbor (KNN) models

Other ML models, such as SVM, random forest, extra tree, and KNN, have been applied to predict response to ICI treatment (178, 179). SVM finds the maximum margin hyperplanes among different data classes, while random forest algorithms perform classification or regression tasks by constructing many decision trees (180). One advantage of random forest is the ability to evaluate the importance of features while training a highly accurate model, and the learning process is fast (181, 182). The extra tree uses a series of decision trees to make final predictions about the class or category to which the data point belongs, but it uses the entire original sample rather than sub-sampling and replacing the data like a random forest (183). KNN is a simple and effective algorithm that finds the K nearest neighbors of a test sample in the feature space and classifies the sample based on the majority class of its neighbors. However, KNN requires high computational time costs and memory requirements, making it less suitable for large datasets (184).

Multiple ML models are often constructed to predict cancer ICI therapy response, and the best-performing model is then chosen to identify potential biomarkers. For example, Shang et al. developed a prediction model for immunotherapy response based on DNA methylation immune scores using lasso regression, SVM, and random forest models, with the lasso regression model performing best (178). Peng et al. developed several classification models based on the gut microbiome dataset from gastrointestinal cancer patients, including elastic net, SVM, random forest, extra tree, and KNN. The accuracy of all models except the SVM model was above 0.9, suggesting that the microbiome has excellent potential as a biomarker to predict the curative effect of PD-1/PD-L1 blockade (185). ML-based approaches have demonstrated outstanding potential in guiding clinical treatment.

#### 3.2.4 Artificial neural network (ANN) and deep learning models

ANN imitates the behavioral characteristics of the biological neural network and uses artificial neurons to form a nonlinear data modeling system. It’s an adaptive information processing system with a learning capacity that can change its internal structure in response to external inputs (186). Multilayer perceptron (MLP) is a forward-structured ANN that utilizes multiple layers of artificial neurons to learn complex nonlinear relationships between input and output data (187, 188). MLP is well-suited for predicting ICI therapy response as it can effectively capture the complex relationship between multiple features and treatment response. Yang et al. constructed an MLP deep learning model that distinguishes treatment responders from non-responders by combining multi-omics data from advanced NSCLC patients treated with PD-1/PD-L1 inhibitors, showing an excellent differentiated performance (165).

In addition to MLP, deep neural networks (DNNs) with two or more hidden layers have better feature representation and complex mapping modeling capabilities than shallow neural networks (189). Peng et al. developed a DNN model that predicts the response to ICI therapy in lung adenocarcinoma patients using somatic mutations. This approach is feasible because there is a strong association between two classifications - durable clinical benefit (DCB) and no durable benefit (NDB) - and various factors such as TMB, neoantigen counts, PD-L1 expression, and the level of immune cell infiltration (190). Arbour et al. constructed a deep natural language processing model to estimate response evaluation criteria in solid tumors that have blocking treatment with PD-1/PD-L1. The model accurately predicted the onset and date of cancer progression and the best overall response (191).

The convolutional neural network (CNN), another type of DNN, is a feedforward neural network that uses convolution calculations and is widely used in computer vision (192, 193). CNN can achieve good data fitting by extracting features through convolutional layers, sharing information in convolutional kernels, and effectively reducing parameters through pooling layers (194). Hu et al. developed a CNN model to predict the impact of PD-1 inhibitor therapy in cancer patients based on hematoxylin and eosin (H&E)-stained images, yielding promising results (195). These findings suggest that deep learning-based models could be integrated into routine clinical practice to screen patients who may benefit from ICI treatment.

#### 3.2.5 Hybrid models of systems biology and machine learning (ML)

The novel integrative computational approach that combines systems biology and ML offers a powerful tool for predicting the therapeutic effects of ICI at the individual level. Przedborski et al. developed a systems biology-informed neural network model that combines both approaches. The model generates simulated clinical data from a systems biology model and extracts patient features to distinguish treatment responders from non-responders using a classification neural network model. The transfer learning strategy is then applied to enhance the model’s predictive ability using actual clinical data (196). The advantage of hybrid models is that they bridge the gap between the two disciplines. Systems biology can generate infinitely simulated data to scale up small clinical datasets, while ML can process multi-omics high-throughput data. Moreover, systems biology models can simultaneously provide mechanistic explanations from a biological perspective that cannot be explained by ML (197).

## 4 Conclusion

Although ICIs have significantly improved cancer treatment, their clinical efficacy remains limited. This underscores the importance of identifying biomarkers to predict patients’ responses to ICI therapy. This review summarizes the advances in biomarker research for ICI treatment, providing a comprehensive classification and overview of these markers. These include tumor cells-derived biomarkers such as TMB, TNB, MSI, and PD-L1 expression, mutated gene biomarkers in pathways, and epigenetic biomarkers. Additionally, the scope encompasses TIME-derived biomarkers, including the immune landscape of TIME biomarkers, inhibitory checkpoints biomarkers, and immune repertoire biomarkers. We also discuss liquid biopsy biomarkers, gut microbiome biomarkers, and metabolomics biomarkers. Currently, clinically used biomarkers include TMB, MSI, and PD-L1 expression. However, these biomarkers often do not effectively predict ICI therapeutic responses in the majority of tumor patients, leading researchers to move beyond the use of a single biomarker and expand the spectrum of explored biomarkers. The article not only elaborates on various biomarkers used to predict tumor ICI treatment response or tolerance and their respective pros and cons but also introduces various laboratory and clinical methods for their detection. Advances in multi-omics sequencing, single-cell, and spatial biology techniques offer a holistic understanding of coordinated anti-tumor systemic immunity. Owing to these advancements, more multi-dimensional ICI response biomarkers have been identified and validated (198–200). Multi-omics sequencing offers a comprehensive insight, allowing a multifaceted evaluation and understanding of the interplay between tumors and the immune system. Single-cell technologies enable the resolution of different types of biomarkers at the individual cell level. Spatial biology techniques reveal cellular spatial distribution within tissue structures, aiding in understanding how biomarkers function within the tumor microenvironment. However, these techniques come with high costs, stringent sample preparation requirements, and complex data analysis. Translating multi-dimensional ICI response biomarker data into clinically meaningful interpretations and applications remains challenging.

Computational prediction models are transforming the way we use clinical biomarkers, a change that is evident not only in current applications but also in ongoing research. These models, by integrating multimodal data related to the immune system and historical knowledge, have established mechanistic models within systems biology aimed at revealing the mechanisms of immunotherapy and identifying key factors for its efficacy (201, 202). Their main advantage lies in the ability to integrate data from different disciplines and previous knowledge, aiding in the explanation and understanding of complex biological system mechanisms. This interdisciplinary integration offers the possibility to discover and validate new biomarkers, as well as to optimize the application of existing ones, thereby advancing personalized medicine. However, challenges such as the complexity of these computational models, the significant demand for computational resources, and the difficulty in accurately estimating model parameters still persist. ML methods have become crucial in the research and clinical application of immunotherapy due to their exceptional data-fitting capabilities and feature engineering. Yet, their predictive logic often lacks transparency, limiting their wider adoption. To address this, there is a push for developing interpretable ML models, which would enhance the explainability and credibility of model predictions and serve as an important research direction. To delve deeper into the dynamic nature of cancer and immune responses, it is necessary to combine mechanistic models in systems biology with advanced computational tools like neural networks and deep learning. The fusion of these approaches can reveal the intrinsic mechanisms of cancer immunotherapy, extracting valuable information and patterns from vast datasets to provide a more comprehensive perspective. This not only aids in making smarter and more adaptive predictions of tumor ICI treatment outcomes, ensuring greater precision, but also provides cancer patients with more accurate and comprehensive dynamic biomarkers, fostering more personalized immunotherapy (160).

Beyond the challenges of model complexity, computational demands, and ML transparency, effectively harnessing vast datasets from diverse sources and ensuring their security pose significant challenges. Addressing the challenge necessitates the establishment of a robust data management platform. Such a platform must ensure the standardization of datasets to facilitate interoperability and maintain stringent privacy safeguards to protect patient information. This infrastructure would serve as a pivotal nexus between the theoretical models developed through research and their practical deployment in clinical settings. By providing a standardized and secure environment for data handling, the platform would not only streamline the adoption of computational models in clinical workflows but also promote the reproducibility of research findings across different institutions.

To successfully establish a data management platform, it’s crucial to coordinate the expertise of professionals from diverse disciplines. Specialists in biology, computing, medicine, and data analysis should collaborate not only to collect and annotate data but also to process and do data quality control, enhancing the utility of the data. Such teamwork is essential for building predictive models for ICI therapies that are meaningful in a real-world medical context. In refining these tools and models, transparency and interpretability are key. The objective is to make both the data and the models understandable, which will increase the credibility of in silico analyses and support their clinical translation. This clarity is essential to ensure that the insights derived from computational research can be confidently applied to inform treatment decisions, thereby bridging the gap between research and clinical application.

Through these measures, we can fully harness the potential of biomarkers and computational models to provide more accurate and personalized treatment options for cancer patients, ultimately achieving the goal of precision medicine. With continuous technological advancements and strengthened interdisciplinary collaboration, the future holds greater breakthroughs in the field of cancer treatment.

## Author contributions

QY: Conceptualization, Investigation, Methodology, Project administration, Writing – original draft, Writing – review & editing. HM: Writing – review & editing. LX: Resources, Writing – review & editing. LS: Funding acquisition, Resources, Supervision, Validation, Writing – review & editing.
