# Corrigendum: Biomarkers and computational models for predicting efficacy to tumor ICI immunotherapy

**DOI:** 10.3389/fimmu.2024.1438587

**Published:** 2024-06-04

**Authors:** Yurong Qin, Miaozhe Huo, Xingwu Liu, Shuai Cheng Li

**Affiliations:** ^1^ Department of Computer Science, City University of Hong Kong, Kowloon, China; ^2^ City University of Hong Kong Shenzhen Research Institute, Shenzhen, Guangdong, China; ^3^ School of Mathematical Sciences, Dalian University of Technology, Dalian, Liaoning, China

**Keywords:** tumor, ICI immunotherapy, biomarkers, computational models, prediction of treatment effectiveness

In the published article, there was an error in the Funding statement. We only stated, “This research was funded by The Science Technology and Innovation Committee of Shenzhen Municipality (Project No. JCYJ20200109143216036),” and omitted “The National Natural Science Foundation of China (Grant No. 62072433)” funding information. The correct Funding statement appears below.

**FUNDING**

The author(s) declare that financial support was received for the research, authorship, and/or publication of this article. This research was funded by The National Natural Science Foundation of China (Grant No. 62072433) and The Science Technology and Innovation Committee of Shenzhen Municipality (Project No. JCYJ20200109143216036).

The authors apologize for this error and state that this does not change the scientific conclusions of the article in any way. The original article has been updated.

