# Supplementary material for: Biomarkers and computational models for predicting efficacy to tumor ICI immunotherapy
Source: Front Immunol. 2024 Mar 8;15:1368749. doi: 10.3389/fimmu.2024.1368749 (PMC10957591; doi:10.3389/fimmu.2024.1368749)
Supplement: Supplementary file 1 [file Table_1.docx]

**Table S1 The detailed information of the biomarkers to predict ICI treatment response.**

| **Biomarker** | **Drug name** | **Cancer name** | **Dataset** | **Detection method** | **Evaluation methods/metrics** | **Advantage** | **Disadvantage** | **Reference** |
| --- | --- | --- | --- | --- | --- | --- | --- | --- |
| TMB | PD-1/PD-L1 | Multiple cancer types including bladder cancer, NSCLC, head and neck cancer and melanoma | 1.Clinical trial data including TMB data and treatment response metrics in PD-1/PD-L1 treated patients.  2.Public bioinformatics databases which include DNA mutation data for TMB study.  3.The published literature provide cancer genomic data for TMB & PD-1/PD-L1 treatment relationship. | NGS (WGS, WES, targeted gene panel sequencing) | Objective response rate (ORR),  Progression-free survival (PFS),  Overall survival (OS),  Response rate (RR) | 1.Pan-cancer potential.  2.High sensitivity: high TMB is often correlated with better response to ICI treatment. | TMB measurement lacks a unified standard, different detection platforms and methods may yield varying results. | (9-12) |
| TNB | PD-1/PD-L1 | Multiple cancer types | 1.Clinical trial data including TNB and treatment response metrics from patients with various types and stages of cancer.  2.The public databases, such as TCGA (The Cancer Genome Atlas) and ICGC (International Cancer Genome Consortium), provide a wealth of multi-omics data including genomic, transcriptomic, and other sequencing data and neoantigen analysis data on various types of cancer.  3.The datasets of published literature include gene mutations, changes in tumor size, and survival time to study TNB & PD-1/PD-L1 responses. | NGS (WGS, WES, targeted gene panel sequencing, RNA-seq), MS | ORR,  PFS,  OS,  RR | 1.Pan-cancer potential.  2.Potential for personalized therapy. | 1.High cost.  2.Complex data analysis. | (24-26) |
| MSI | PD-1/PD-L1 | Multiple cancer types | 1.Clinical trial data including MSI test results and treatment response data from patients with various types and stages of cancer.  2.Public databases including MSI data or clinical data such as cancer type, survival status, etc.  3.Published literature including data on MSI status and treatment response. | IHC,  PCR,  NGS (WGS/WES/Gene panel sequencing ) | ORR,  PFS,  OS,  RR | 1.Pan-cancer potential.  2.The current MSI testing technology is well-established and can provide patients with accurate and quick information. | 1.The determination of MSI may involve more complex and expensive testing techniques.  2.Interpretation challenges. | (39,40) |
| PD-L1 expression | PD-1/PD-L1 | Multiple cancer types | 1.Clinical trial data including patient information, PD-1/PD-L1 treatment regimen and treatment response data, PD-L1 expression levels.  2.Public databases: PD-L1 expression from cancer samples & its correlation with PD-1/PD-L1 treatment response.  3.Published literature: the relationship between PD-L1 expression levels and treatment outcomes. | IHC | ORR,  PFS,  OS,  RR | 1.Pan-cancer potential.  2.Clinical relevance: PD-L1 expression is associated with response to PD-1/PD-L1 therapy. | The cost is high, there is a lack of standardized testing methods, interpretation is challenging. | (50-52) |
| Mutated gene biomarkers in pathways | PD-1/PD-L1 | Multiple solid cancers | 1.Whole-genome/exome sequencing data: information on gene mutations in the patient's cancerous tissue.  2.Functional pathway analysis data: identification of biological pathways or networks involved in the patient's mutations.  3.The therapeutic response data: changes in tumor size or aggressiveness before and after treatment, and the correlation between specific gene mutations or pathways and treatment response.  4.Clinical data：details of the patient's medical history, treatment regimen, drug dosages, and treatment cycles. | NGS,  PCR,  IHC,  ISH | ORR,  PFS,  OS,  RR | 1.Multiple applications: they can be relevant across different types of cancers and therapies, not limited to just PD-1/PD-L1 inhibitors.  2.Targeted insight: understanding specific mutated pathways can provide targeted insights into treatment resistance and susceptibility. | 1.Complex analysis.  2.High cost.  3.Time-consuming.  4.Limited availability: the expertise and technology required for such intricate analyses may not be readily available in all healthcare settings. | (58-67) |
| Epigenetic biomarkers | PD-1/ PD-L1 | Multiple solid cancers | 1.DNA methylation data.  2.Histone modification data.  3.Chromatin remodeling data.  4.Public database information: these databases provide vast epigenetic data on cancers.  5.Epigenetic changes related to treatment. | DNA methylation sequencing (WGBS, RRBS, MeDIP, DNA methylation chip,MS-PCR),  Histone modifications (CHIP-seq, ELISA, Western blot) | ORR,  PFS,  OS,  RR | 1.Dynamic response: epigenetic markers might more dynamically reflect the status of tumors and the immune microenvironment, as they are reversible.  2.Personalized treatment. | The detection and analysis of epigenetic biomarkers may involve more complex and expensive technologies, and there may currently be a lack of unified standards and assurances of accuracy. | (80-86) |
| Immune landscape of TIME biomarkers | PD-1/PD-L1 | Multiple solid cancers | Data sources and types:  bulk sequencing: this includes transcriptomic and methylation data. It offers a holistic view derived from a large number of cells.  Single-cell sequencing: allows for the assessment of TIME at a single-cell level, offering researchers a high-resolution view, enabling the identification of novel tumor-associated immune cell subtypes.  Spatial transcriptome sequencing: enables researchers to study the spatial distribution characteristics of cells within the TIME. | Flow cytometry and IHC，NGS(bulk sequencing, such as transcriptome and methylation sequencing, single-cell sequencing, and spatial transcriptome sequencing) | ORR,  PFS,  OS,  RR | 1.Pan-cancer potential.  2.Comprehensive evaluation of the tumor immune response, covering aspects like cell type, abundance, location, and function.  3. High resolution through single-cell sequencing techniques, allowing for a detailed understanding of the TIME.  4.Spatial transcriptomics provides insights into the spatial distribution of cells within the TIME, crucial for predicting immune therapy responses.  5.Diverse data analysis tools available for transcriptome and methylation data, offering greater flexibility for researchers. | 1.High costs and limited accessibility for some technologies, especially advanced sequencing methods.  2.Tissue sample availability might be a limitation for techniques like flow cytometry and IHC.  3.Complex data sets require advanced data processing and analytical skills, which might be a challenge for some researchers. | (87-90) |
| Inhibitory checkpoints biomarkers | PD-1/PD-L1 | Multiple solid cancers | The gene and protein expressions of Inhibitory checkpoint biomarkers, such as TIM-3 and LAG-3. | Flow Cytometry, IHC, Western blot, Real-time quantitative PCR (qPCR), NGS(WGS,WES,RNA-seq) | ORR,  PFS,  OS,  RR | 1.Pan-cancer potential.  2.Potential for combinatorial therapies: recognizing the presence of multiple inhibitory checkpoints can lead to combination therapies targeting both PD-1/PD-L1 and other checkpoints, potentially increasing the likelihood of therapeutic response. | 1.Increased complexity and cost of assessment.  2.Interpretation of results can be complicated. | (61,93,94) |
| Immune repertoire biomarkers | anti-CTLA-4, anti-PD-1/PD-L1 | Multiple solid cancers | Clinical trial data: patient data pre/post PD-1/PD-L1 treatment, including TCR/BCR diversity, dominant clones, and repertoire changes. Additionally, there's the patient's post-treatment response. | Immune repertoire sequencing | ORR,  PFS,  OS,  RR | Reflects the diversity of the immune response, providing a comprehensive overview of the patient's immune status. | 1.Complexity: the diversity of T-cell and B-cell receptors in the adaptive immune system presents technical challenges.  2.Standardization Issues: different platforms might produce varying results. | (95-97) |
| Liquid biopsy biomarkers | PD-1/PD-L1 | Multiple solid cancers | 1.Clinical trial data: levels or presence status of biomarkers, treatment response and outcomes of patients.  2.Public databases: dynamic changes of biomarkers, treatment histories and treatment response of patients.  3.Published literature: relationships between biomarkers and therapeutic outcomes. | Quantitative PCR, ddPCR, CellSearch, FISH and NGS (Targeted deep sequencing, untargeted sequencing) | ORR,  PFS,  OS,  RR | 1.Non-invasive.  2.Real-time monitoring capability.  3.Suitable for early detection.  4.Less risky than traditional biopsies. | 1.Potential for false positives and false negatives.  2.Might not capture the full spectrum of tumor mutations.  3.Limited to detectable biomarkers in circulation.  4.Technological and standardization challenges. | (104,105) |
| Gut microbiome biomarkers | PD-1/PD-L1 | Multiple solid cancers | 1.Clinical trial data: microbial community profiles pre and post-treatment,  clinical treatment outcomes and metrics.  2.Public databases or databases from published literature  provide vast microbial sequencing datasets from diverse populations. | PCR, targeted NGS such as 16S rRNA sequencing, non-targeted NGS, MS | ORR,  PFS,  OS,  RR | 1.Personalized medicine.  2.Guides treatment strategies.  3.Non-invasive. | 1.Complexity.  2.Result variability.  3.Causal relationship not fully established.  4.Further validation needed. | (128,129) |
| Metabolomics biomarkers | PD-1/PD-L1 | Multiple solid cancers | 1.Metabolite data: acquired from patient samples using liquid chromatography-mass spectrometry (LC-MS) or gas chromatography-mass spectrometry (GC-MS).  2.Clinical data: patient baseline information, treatment types, and outcome metrics.  3.Bioinformatics databases: information on genes, enzymes, or pathways associated with metabolites.  4.Public databases: provide metabolite data.  5.Literature: known data on the relationship between metabolites and PD-1/PD-L1 response. | NMR (nuclear magnetic resonance), MS (LC-MS, GC-MS ) | ORR,  PFS,  OS,  RR | 1.Due to the involvement of multiple metabolites, it might offer a more comprehensive prediction of treatment efficacy.  2.Relatively non-invasive with fewer side effects and better comfort. | 1.Numerous metabolites, requiring big data and algorithms for key indicator selection.  2.High cost and technical threshold, not all medical institutions can perform it. | (139-141) |
