# Supplementary material for: Biomarkers and computational models for predicting efficacy to tumor ICI immunotherapy
Source: Front Immunol. 2024 Mar 8;15:1368749. doi: 10.3389/fimmu.2024.1368749 (PMC10957591; doi:10.3389/fimmu.2024.1368749)
Supplement: Supplementary file 2 [file Table_2.docx]

**Table S2 The detailed information of the mechanistic models to predict ICI treatment response.**

| **Model type** | **Drug**  **name** | **Cancer type** | **Dataset** | **Prediction target** | **Evaluation methods/metrics** | **Biomarkers** | **Code/software** | **Advantage** | **Disadvantage** | **Reference** |
| --- | --- | --- | --- | --- | --- | --- | --- | --- | --- | --- |
| PK/PD Model | Atezolizumab (anti-PD‐L1) | NSCLC | 88 patients from PCD4989g phase I clinical trial | Change in tumor size (the sum of the longest diameter) | AUC,  Relative change from baseline,  Relative standard errors of prediction | Two plasma biomarkers, ITAC and IL-18 | R script | Provides early insights into drug efficacy and safety | Model construction and validation require a substantial amount of data and expertise | (155) |
|  | Nivolumab (anti-PD-1) | Various solid tumor types | Patient data from various solid tumor types, including clinical trials and published studies | To explore the relationship between the time-varying clearance of nivolumab and disease dynamics, and to analyze its implications on exposure response analysis | Pharmacokinetic (PK) metrics: Time-varying clearance,  especially in relation to post-treatment disease status | Longitudinal tumor size | R (version 3.3.0) |  |  | (156) |
| PDE model | Anti-PD‐L1, anti-TGF-β | Not specific | Mouse experimental data, Tregs under treatment with anti-PD-1 data | Effectiveness of TGF-β inhibitor in overcoming primary resistance to PD-1 blockade. | Simulation of tumor volume hyperprogression, Parameter sensitivity analysis for tumor volume | Two cancer-specific parameters | Python 3.5.4 | PDE models allow for spatiotemporal dynamics and complex interactions, enhancing prediction of therapy effectiveness. | PDE models are computationally intensive, require many parameter estimates, and pose validation challenges. | (158) |
| Signal networks-based model | Pembrolizumab (anti-PD-1) | NSCLC | 34 stage IV NSCLC patients | Clinical responses to PD-1 immunotherapy, based on PD-L1 expression, chemokines, and immunosuppressive molecules profiles. | Comparison of match scores between non-responders and responders  Correlation between predicted and reported patient clinical responses | 24 chemokines and immunosuppressive molecules | Weka 3 (Java) | These models enhance prediction accuracy by capturing protein-protein interactions and integrating patient-specific genetic data. | They require detailed genetic data and may oversimplify biological complexities, potentially limiting accuracy. | (159) |
| QSP model | Nivolumab(anti-PD-1) | TNBC | WES and scRNA-seq data from TNBC patients | Immunotherapy response | Accuracy on treatment response  Comparison of simulation-derived tumor mixing scores with clinical data | Density of CD8^+^ T cells and the ratio of CD8^+^ T to foxp3+ T cells | C++ | Capable of multiscale analysis, enabling detailed exploration of biomarkers across various temporal and spatial scales. | High complexity requiring extensive resources and expertise; validation can be challenging due to multiscale considerations. | (161) |
| ABM | anti-PD-1 | Glioblastoma (GBM) | A three-dimensional ABM was used to simulate the interactions between different populations of immune cells, oncolytic Herpes Simplex Virus, and anti-PD-1 immunotherapy in the treatment of GBM | The efficacy of the treatment outcome, with a primary focus on the reduction in tumor size and effective immune response | The primary evaluation method is a three-dimensional ABM of GBM, which assesses the interactions between immune cells and the oncolytic virus. The study determines the efficacy of different dosing strategies by simulating the spread and effect of the virus on the tumor. | -- | MATLAB | Spatial interaction simulation: ABMs are commonly used to simulate three-dimensional spatial interactions at the tissue or cell level, which is especially important when considering interactions between cells. | Computational complexity:Agent-based simulations typically require a significant amount of computation, especially when the number of cells or tissues being simulated is very large. | (164) |
