# Supplementary material for: Biomarkers and computational models for predicting efficacy to tumor ICI immunotherapy
Source: Front Immunol. 2024 Mar 8;15:1368749. doi: 10.3389/fimmu.2024.1368749 (PMC10957591; doi:10.3389/fimmu.2024.1368749)
Supplement: Supplementary file 3 [file Table_3.docx]

**Table S3 The List of machine learning models to predict ICI treatment response.**

| **Machine learning models** | **Drug name** | **Cancer name** | **Dataset** | **Input** | **Output** | **Evaluation methods/metrics** | **Software name/Predictor** | **Code/software availability** | **Advantage** | **Disadvantage** | **Reference** |
| --- | --- | --- | --- | --- | --- | --- | --- | --- | --- | --- | --- |
| Lasso regression | Anti-PD-1/PD-L1 | 18 cancer types | Literature,  TCGA,  GEO | DM | ORR | LOOCV, AUC | DNA methylation biomarkers | Lasso function in MATLAB | 1.Prevents overfitting: By adding the L1 penalty term, lasso regression can help reduce overfitting.  2.Model simplification: Since lasso may result in a weight of zero for some features, it means the model might be simpler and easier to interpret. | 1.Feature selection might be overly aggressive.  2.Instability.  3.Doesn't handle cases where number of features exceeds number of samples well. | (170) |
| Linear elastic-net | Anti-PD-1/PD-L1 | Advanced  solid tumors | MOSCATO,  The immune phenotype dataset,  Immunotherapy-treated dataset,  TCGA,  TCIA | Computed tomography (CT) scans (DICOMs),  RNA-seq data,  pathology slides，  clinical data | Res/Non-Res | CDV, AUC | Radiomic signature | R package glmnet (version 2.0-10) | Flexibility: With its regularization parameters, elastic-net can balance between pure lasso and pure ridge, making it more adaptive in various application scenarios. | Computational complexity: Compared to lasso and ridge, the computations for elastic-net are generally more complex and time-consuming. | (171) |
| Logistic regression | Anti-PD-1/PD-L1 | Urothelial carcinoma | One clinical dataset | CT,  clinical features | High-risk/low-risk | CDV, AUC | Radiomics-based model | Using R statistical software | 1.Simplicity & intuitiveness: Logistic regression is a simple model that's easy to understand and interpret.  2.Efficiency: It's computationally efficient, especially when the number of features isn't large. | 1. Linear decision boundary: Logistic regression assumes a linear decision boundary, which might not be suitable for complex problems where the decision boundary is nonlinear.  2.Sensitive to outliers: Outliers can affect the performance of the model. | (175) |
| Logistic regression | Anti-PD-1/PD-L1 | Advanced cancer | One clinical dataset | SNPs | Res/Non-Res | KFCV, AUC | Elastic-net penalized logistic regression | R package glmnet | 1.Versatility: the logistic regression model is a commonly used classification algorithm suitable for various types of datasets, including the analysis of the association between gene polymorphism and treatment response.  2.Ease of interpretation: the model's output is probability values, which can be easily interpreted as the likelihood of an event occurring, making it helpful for explaining the relationship between gene polymorphism and treatment efficacy. | 1.Computationally intensive: elastic net requires tuning of two hyperparameters (α for the mix of L1/L2 regularization and λ for the overall strength of the regularization). This can make the process computationally intensive, especially in a grid search for the best hyperparameters.  2.Data standardization requirement: the predictors usually need to be standardized (to have mean zero and variance one) before applying the elastic net because the penalty term is sensitive to the scale of predictors. | (176) |
| Lasso logistic regression | Anti-PD-1 | Nasopharyngeal carcinoma | One clinical dataset | Clinical features of serum biomarkers | High-risk/low-risk | KFCV, AUC | Lasso logistic regression | R foundation for statistical computing | 1.Using a dynamic monitoring approach and utilizing LASSO regression to construct a prediction model that better reflects the patient's disease status and treatment effects.  2.Developing a risk score prediction model based on two markers screened by Lasso logistic regression, which has good predictive performance (AUC=0.737) and can effectively predict the efficacy of PD-1 inhibitors.  3.The model can also predict patient survival, which is superior to the traditional TNM staging prediction model and can provide a basis for patient treatment and disease monitoring. | 1.This is a single-center retrospective analysis and further validation in multi-center prospective studies is needed.  2. The study only selected two serum markers as the basis for the risk score prediction model, and other more representative biomarkers may have been overlooked.  3.Although the predictive performance of the model is good, it still needs to take into account patient heterogeneity and various factors that may affect the model's prediction results during practical application. | (177) |
| Lasso regression, random forest and SVM | Anti-PD-1/PD-L1 | NSCLC | GEO,  TCGA | DM | Res/Non-Res | KFCV, AUC | MeImmS | R package glmnet, randomForest, and e1071 | 1.Specificity to DNA methylation: The MeImmS focuses on DNA methylation of 8 CpG sites, offering a targeted approach to understanding the genetic factors influencing immunotherapy response.  2.Regulation of immune-related genes: The chosen CpG sites regulate the expression of immune-related genes, implying a direct link between the markers and the immune response. | 1.Although the article emphasizes the effectiveness of MeImmS, it does not provide comparison data with other existing biomarkers or clinical variables, making it difficult to determine its superiority.  2.The article does not provide specific recommendations on how to apply MeImmS in clinical practice, such as how to interpret the results of MeImmS or how to guide immunotherapy based on MeImmS. | (178) |
| Random forest, extra trees, SVM, elastic net, and KNN | Anti-PD-1/PD-L1, anti–  CTLA-4 | Gastrointestinal cancer, Melanoma | Three clinical datasets | 16S rRNA sequencing data | Res/Non-Res | KFCV | 16S-rRNA–derived microbiome profiles | Scikit-learn python package | 1.Comprehensive analysis: the study undertook a comprehensive analysis, which ensures that various facets of the gut microbiomes were examined.  2.Dynamic sampling: the collection of fecal samples both prior to and during immunotherapy allows for the observation of dynamic changes in the gut microbiome in response to treatment. | Although the authors mentioned that they identified specific bacteria species and functions related to treatment response, they did not study how these microorganisms affect the immune response of patients. This is an important gap, because understanding how these microorganisms affect the immune response can help us better understand how they affect the response to PD-1/PD-L1 treatment. | (185) |
| Multiple layer perceptron (MLP) | Anti-PD-1/PD-L1 | Advanced stage NSCLC | One clinical dataset,  four  web datasets | Radiomic features,  Blood test data,  Patient clinical data | Res/Non-Res | KFCV, AUC | MLP | Deep neural network (PyTorch 1.2) | 1.Integrating multi-modal data: The model amalgamates various types of data, including radiomics from CT scans, laboratory data, and baseline clinical information, providing a comprehensive view of the patient's condition.  2.Temporal attention mechanism: This model employs the simple temporal attention (SimTA) module to handle asynchronous time-series imaging and laboratory data, effectively capturing crucial temporal information associated with disease progression.  3.Surpassing traditional assessment methods: Compared to the conventional RECIST evaluation method, this model offers a superior differentiation of survival benefits in patients with stable disease. | 1.Complexity of asynchronous data processing: Handling asynchronous time-series data might pose technical challenges, and at times, may not be sufficient to capture all pivotal clinical variations.  2.Model validation: Although the model has undergone cross-validation, for broader applicability, it may further require validation in other independent datasets or a more diverse patient cohort. | (165) |
| Deep neural network  (DNN) | Anti-PD-1/PD-L1,  anti-CTLA4 | Lung adenocarcinoma | TCGA  Cohort,  TSP cohort,  Broad cohort,  Three clinical  cohorts | Somatic mutations | Immune DCB / NDB | CDV | DNN | TensorFlow library (PYTHON 3.6.3) | 1.Multimodal data analysis: the model takes into account a variety of data types, including immune cell infiltration, PD-L1 expression, and TMB, providing a holistic view of the factors influencing ICB therapy efficacy.  2.Comprehensive biomarker analysis: the model doesn't just rely on one or two markers; it examines a wide range of factors, from TMB and neoantigens to transversion or transition rates, making its predictions more robust. | 1.Need for external validation: While the DNN was validated in three cohorts, its generalizability to broader populations remains to be seen. External validation in diverse populations would further validate the model's efficacy.  2.Model interpretability: DNNs, being a form of black-box model, can sometimes be difficult to interpret in terms of which features are most important for predictions. This can pose challenges in understanding the biological implications of the model's predictions. | (190) |
| Deep Learning | Anti-PD-1 | NSCLC | MSK | Clinical text reports | Res/Non-Res | RRSSCV | DL model | In Python v3.7.1 using TensorFlow | 1.Objective Assessment: The deep learning model is trained to use radiology text reports to estimate the gold-standard RECIST-defined results, providing a reliable and replicable way to assess the effects of specific treatments.  2.Accuracy: After training and testing using text reports from non-small cell lung cancer patients, the model can accurately estimate the best overall response and progression-free survival rates. | 1.Data Dependency: The model relies on radiology text reports, implying its efficacy and accuracy largely depend on the quality and consistency of the reports.  2.Generality: The model was trained for a specific type of cancer and treatment method (PD-1 blockade), which might limit its applicability in other cancers or treatment modalities. | (191) |
| Convolutional neural network (CNN) | Anti-PD-1 | Melanoma,lung cancer | TCGA-SKCM database | H&E images | Res/Non-Res | KFCV, AUC | CNN | Scikit-learn in Python | 1.Innovative Application: This model is the first to utilize H&E images for predicting immunotherapy responses, paving the way for new research in the field.  2.Promising Results in Melanoma Samples: With 54 melanoma H&E samples, the model achieved an AUC of 0.778, indicating good predictive performance for this specific cancer type. | Variable Performance Across Cancers: While the model performed well on melanoma samples, its efficacy was somewhat diminished in lung cancer samples. This suggests that the model may not be universally effective for all cancer types. | (195) |
| Systems biology-informed neural networks (SBINN) | Anti-PD-1 | Head and neck squamous cell carcinoma (HNSCC) | One clinical dataset,  Simulation dataset | Clinical data,  selected features | Res/Non-Res | KFCV, AUC | SBINN | <https://github.com/perwu/MIAS> | 1.Integrative approach: Combining systems biology with machine learning offers a holistic view of patient-specific biology, allowing for a deeper understanding of the dynamics between the immune system and the tumor.  2.Transfer learning: Leveraging transfer learning with simulated clinical data enhances the prediction accuracy of the SBINN, making the model more reliable. | 1. Complexity: The integrative approach might be computationally intensive and require more data preprocessing than traditional models. 2.Dependency on simulated data: While transfer learning improves prediction accuracy, the model's reliance on simulated clinical data might not always capture real-world complexities. | (196) |

AUC, Area under the curve; CDV,  cross-dataset validation; DCB / NDB, durable clinical benefit/no durable benefit; DICOMs, Digital Imaging and Communications in Medicine; DL model, deep natural language processing model; DM, DNA methylation; KFCV,  K-fold cross validation; LIP-SVM, liquid immune profiling-SVM; LOOCV,  leave-one-out cross validation; MPR/ No-MPR, major pathological response/no- major pathological response; Res/Non-Res,  responder/non-responder; RRSSCV,  repeated random sub-sampling cross validation.
